# Prevalence of asymptomatic Zika virus infection: a systematic review

**DOI:** 10.2471/BLT.17.201541

**Published:** 2018-04-27

**Authors:** Michelle M Haby, Mariona Pinart, Vanessa Elias, Ludovic Reveiz

**Affiliations:** a Department of Chemical and Biological Sciences, Universidad de Sonora, Blvd Encinas y Rosales S/N, Colonia Centro, C.P. 83000, Hermosillo, Sonora, Mexico.; b Cochrane Skin Group, The University of Nottingham, Nottingham, England.; c Pan American Health Organization, Washington, United States of America.

## Abstract

**Objective:**

To conduct a systematic review to estimate the prevalence of asymptomatic Zika virus infection in the general population and in specific population groups.

**Methods:**

We searched PubMed®, Embase® and LILACS online databases from inception to 26 January 2018. We included observational epidemiological studies where laboratory testing was used to confirm positive exposure of participants to Zika virus and in which Zika virus symptom status was also recorded. We excluded studies in which having symptoms of Zika virus was a criterion for inclusion. The main outcome assessed was percentage of all Zika virus-positive participants who were asymptomatic. We used a quality-effects approach and the double arcsine transformation for the meta-analysis.

**Findings:**

We assessed 753 studies for inclusion, of which 23 were included in the meta-analysis, totalling 11 305 Zika virus-positive participants. The high degree of heterogeneity in the studies (*I^2^* = 99%) suggests that the pooled prevalence of asymptomatic Zika virus-positive participants was probably not a robust estimate. Analysis based on subgroups of the population (general population, returned travellers, blood donors, adults with Guillain–Barré syndrome, pregnant women and babies with microcephaly) was not able to explain the heterogeneity. Funnel and Doi plots showed major asymmetry, suggesting selection bias or true heterogeneity.

**Conclusion:**

Better-quality research is needed, using standardized methods, to determine the true prevalence of asymptomatic Zika virus and whether it varies between populations or over time.

## Résumé

**Objectif:**

Réaliser une revue systématique afin d'estimer la prévalence des infections à virus Zika asymptomatiques dans la population générale et dans des groupes de population spécifiques.

**Méthodes:**

Nous avons fait des recherches en ligne dans PubMed®, Embase® et LILACS afin de trouver des références parues depuis la date de création de ces bases de données jusqu'au 26 janvier 2018. Nous avons inclus des études épidémiologiques observationnelles dans lesquelles des tests en laboratoire ont été utilisés pour confirmer l'exposition des participants au virus Zika et dans lesquelles le statut symptomatique/asymptomatique de l'infection à virus Zika a été consigné. Nous avons écarté les études pour lesquelles l'existence de symptômes d'infection à virus Zika a été utilisé comme critère d'inclusion. Le principal résultat évalué a été le pourcentage de participants asymptomatique par rapport à l'intégralité des participants infectés par le virus Zika. Pour notre méta-analyse, nous avons employé une approche qualité-effets et réalisé une transformation à double arc-sinus.

**Résultats:**

Pour l'inclusion dans notre méta-analyse, nous avons évalué 753 études, 23 d'entre elles ont été retenues, ce qui représente 11 305 participants infectés par le virus Zika. Compte tenu de la très grande hétérogénéité des études (*I^2^* = 99%), la prévalence groupée des participants asymptomatiques infectés par le virus Zika ne constitue probablement pas une estimation fiable. L'analyse portant sur des sous-groupes de population (population générale, personnes revenant de voyages, donneurs de sang, adultes atteints du syndrome de Guillain-Barré, femmes enceintes, nouveau-nés présentant une microcéphalie) n'a pas permis d'expliquer cette hétérogénéité. Les courbes de biais (*«funnel plot»* et *«Doi plot»*) ont révélé une asymétrie majeure, suggérant ainsi un biais de sélection ou une vraie hétérogénéité.

**Conclusion:**

Des études de meilleure qualité doivent être réalisées en utilisant des méthodes standardisées afin de déterminer la véritable prévalence des infections asymptomatiques à virus Zika et de déterminer si elle varie entre les populations ou au fil du temps.****

## Resumen

**Objetivo:**

Llevar a cabo una revisión sistemática para estimar la prevalencia de una infección asintomática del virus de Zika en la población general y en grupos de población específicos.

**Métodos:**

Se realizaron búsquedas en las bases de datos en línea de PubMed®, Embase® y LILACS desde el origen hasta el 26 de enero de 2018. Se incluyeron estudios epidemiológicos observacionales en los que se usaron pruebas de laboratorio para confirmar la exposición positiva de los participantes al virus de Zika y en las que también se registró el estado de los síntomas del virus de Zika. Se excluyeron los estudios en los que mostrar síntomas del virus de Zika fue un criterio de inclusión. El principal resultado evaluado fue el porcentaje de todos los participantes que resultaron positivos al virus de Zika y no presentaban síntomas. Se siguió un enfoque calidad-efectos y la transformación de arcoseno doble para el metanálisis.

**Resultados:**

Se evaluaron 753 estudios para su inclusión, de los cuales 23 se incluyeron en el metanálisis, sumando un total de 11 305 participantes positivos para el virus de Zika. El alto grado de heterogeneidad en los estudios (*I^2^* = 99%) sugiere que la prevalencia combinada de participantes asintomáticos con el virus de Zika probablemente no era una estimación robusta. Los análisis basados en subgrupos de población (población general, viajeros de vuelta, donantes de sangre, adultos con síndrome de Guillain-Barré, mujeres embarazadas y bebés con microcefalia) no pudieron explicar la heterogeneidad. Los gráficos de Funnel y Doi mostraron una asimetría importante, lo que sugiere un sesgo de selección o una verdadera heterogeneidad.

**Conclusión:**

Se necesita una investigación de mejor calidad, que use métodos estandarizados, para determinar la verdadera prevalencia del virus de Zika asintomático y si varía entre las poblaciones o con el tiempo.

## الملخص

**الغرض:**

إجراء مراجعة منهجية لتقدير انتشار عدوى فيروس زيكا غير المصحوب بظهور أعراض بين عموم السكان وبين قطاعات بعينها من السكان.

**الطريقة:**

قمنا بالبحث في قواعد بيانات Embase‎® وPubMed‎® وLILACS الموجودة على شبكة الإنترنت منذ إنشائها وحتى 26 يناير/كانون الثاني 2018. وقمنا بتضمين دراسات رصدية وبائية والتي تم فيها استخدام التجارب المختبرية للتأكيد على تعرض المشاركين إلى فيروس زيكا والتي تم فيها أيضًا تسجيل حالة أعراض الفيروس. وقمنا باستبعاد الدراسات التي كانت فيها الإصابة بأعراض بفيروس زيكا معيارًا للمشاركة. وقد كان التقييم الرئيسي للنتائج هو النسبة المئوية لجميع المشاركين الذين تأكدت إصابتهم بفيروس زيكا دون ظهور أعراض عليهم. واستخدمنا نهج المؤشرات النوعية كما استخدمنا تحويل الأرسين المزدوج لإجراء التحليل التلوي.

**النتائج:**

قمنا بتقييم 753 دراسة لتضمينها منها 23 دراسة تم تضمينها في التحليل التلوي، بإجمالي 11305 من المشاركين الذين تأكدت إصابتهم بفيروس زيكا. وكانت النسبة عالية من عدم التجانس في تلك الدراسات (حيث بلغ مربع معامل عدم التجانس ‏I ^2 ^: = 99‏٪) مما يشير إلى أن الانتشار المجمع للمشاركين الذين تأكدت إصابتهم بفيروس زيكا دون ظهور أعراض لم يكن في الغالب متقن التقدير.

لم يتمكن التحليل القائم على القطاعات الفرعية من السكان (عموم السكان، المسافرون العائدون، المتبرعون بالدم، الأشخاص البالغون المصابون بمتلازمة جيان باري، النساء الحوامل والأطفال الرضع المصابون بتشوهات صِغر حجم الرأس) من تفسير عدم التجانس. ولقد أظهر مخطط "دوي" والمخطط القُمعي تفاوتًا كبيرًا، مما يشير إلى الانحياز في الاختيار أو عدم التجانس الواقعي.

**الاستنتاج:**

توجد حاجة إلى القيام بأبحاث ذات جودة أفضل، واستخدم طرق موحدة، لكي يتم تحديد الانتشار الفعلي لفيروس زيكا غير المصحوب بظهور أعراض وما إذا كان الفيروس يختلف باختلاف السكان أو بمرور الوقت.

## 摘要

**目的:**

对一般人群和特定人群中无症状寨卡病毒感染的患病率进行系统评审。

**方法:**

自 2018 年 1 月 26 日起，我们检索了 PubMed®、Embase® 和 LILACS 的在线数据库。我们纳入观察性流行病学研究，使用实验室测试确认参与者接触寨卡病毒的测试结果为阳性并且还记录了塞卡病毒症状状态。我们排除将塞卡病毒症状作为纳入标准的研究。主要评估结果是所有无症状的塞卡病毒阳性参与者的百分比。我们使用质量效应方法和双反正弦变换进行元分析。

**结果:**

我们评估了 753 项研究，其中 23 项被纳入元分析，共计 11305 位塞卡病毒阳性参与者。研究中的高度异质性 （*I^2^* = 99%） 表明，无症状塞卡病毒阳性参与者的综合患病率可能不是抗差估计。基于人口亚群（一般人群、返回旅行者、献血者、患有格林-巴利综合征成年人、孕妇和小头畸形婴儿）的分析无法解释异质性。漏斗图和数字对象标识符图显示了主要的不对称，表明选择性偏差和真正的异质性。

**结论:**

需要使用标准方法的更高质量研究来确定无症状塞卡病毒真正的患病率以及它是否会在人群之间或随着时间而变化。****

## Резюме

**Цель:**

Провести систематический обзор для оценки распространенности бессимптомной инфекции, вызываемой вирусом Зика, в общей популяции и в определенных группах населения.

**Методы:**

Авторы провели поиск в онлайн-базах данных PubMed®, Embase® и LILACS с момента их создания до 26 января 2018 года. В обзор были включены наблюдательные эпидемиологические исследования, в которых использовалось лабораторное тестирование для подтверждения инфицирования участников вирусом Зика, а также те, в которых было указано наличие или отсутствие проявления симптомов этой инфекции. Авторы исключили исследования, в которых наличие симптомов этой инфекции было критерием для включения. Основной результат оценивался как процентная доля участников с положительным результатом обследования на вирус Зика, у которых инфекция, определенная по отношению к общему числу участников, протекала бессимптомно. Для метаанализа авторы использовали подход, основанный на качественном эффекте, и двойное арксинус-преобразование.

**Результаты:**

Авторы провели оценку 753 исследований, из которых 23 были включены в метаанализ, т. е. в общей сложности 11 305 участников, инфицированных вирусом Зика. Высокая степень гетерогенности в исследованиях (*I^2^* = 99%) свидетельствует о том, что общая распространенность участников, инфицированных вирусом Зика, у которых отсутствовали проявления симптомов инфекции, по-видимому, являлась недостоверной оценкой. Анализ, основанный на подгруппах населения (общая популяция, вернувшиеся из поездок путешественники, доноры крови, взрослые с синдромом Гийена — Барре, беременные женщины и дети с микроцефалией), не смог объяснить эту гетерогенность. Воронкообразная диаграмма и диаграмма ЦИО показали большую асимметрию, что свидетельствует о систематической ошибке отбора или истинной гетерогенности.

**Вывод:**

Необходимо провести более качественные исследования с использованием стандартизированных методов для определения истинной распространенности бессимптомной инфекции, вызываемой вирусом Зика, а также выяснить, изменяется ли она среди популяций или с течением времени.****

## Introduction

By 25 May 2017, 48 countries and territories in the Americas had confirmed autochthonous, vector-borne transmission of Zika virus disease and 26 had reported confirmed cases of congenital syndrome associated with the infection.^1^ Symptoms are often very mild or not present. When symptomatic, the infection may include rash, fever, arthralgia and conjunctivitis. Zika virus infection during pregnancy is a cause of congenital Zika syndrome^2^ and it may also be a trigger for Guillain‒Barré syndrome.^2^^,^^3^

It has been widely reported that approximately 80% of people with Zika virus infection are asymptomatic. This statement is based on a household survey on Yap State in 2007^4^ that has been cited in many publications on Zika virus. Among 557 residents who provided blood samples, 414 had immunoglobulin (Ig) M antibody against Zika virus and 156 of these (38%) reported an illness that met the definition for suspected Zika virus disease. However, 27 (19%) of the 143 residents who had no detectable IgM antibody against Zika virus also reported an illness that met the definition for suspected Zika virus disease. The authors concluded that, among participants who had IgM antibody against Zika virus, a total of 19% (38% minus 19%) had symptoms that were likely due to the Zika virus infection. When adjusted to the total Yap population aged 3 years or older, the authors estimated that 18% of those infected (95% confidence interval, CI: 10‒27%) had a clinical illness that was probably attributable to Zika virus. From these data we, and other authors, concluded that 82% of the population infected with Zika virus were asymptomatic.

Lack of signs and symptoms of Zika virus infection does not necessarily imply protection from potential complications, such as microcephaly in babies and Guillain‒Barré syndrome in adults. This has implications for surveillance, treatment and research efforts. For example, an analysis was conducted of pregnancies completed between 15 January and 22 September 2016, and recorded in the United States Zika pregnancy registry.^5^ Among women with laboratory evidence of Zika virus infection, there was no difference in the prevalence of birth defects in babies born to asymptomatic (16/271, 6%; 95% CI: 4–9%) or symptomatic women (10/167, 6%; 95% CI: 3–11%). Thus, if the asymptomatic pregnant women had not been included in Zika virus surveillance the 16 babies born with birth defects may not have been attributed to Zika virus.

Currently, with the exception of asymptomatic pregnant women, only people with suspected infection (i.e. symptomatic) generally undergo laboratory testing for Zika virus infection as part of national surveillance efforts.^6^ Thus, the true prevalence of infection and related complications is likely to be underestimated and biased towards those who seek care or develop a viral disease in response to infection.^7^ Knowing the prevalence of asymptomatic Zika virus infection is important for assessing the effectiveness and cost‒effectiveness of interventions, including vaccines, to prevent or treat infection. The prevalence is also needed for decision-making about the value of scaling-up surveillance efforts.

The aim of the current review was to estimate the prevalence of asymptomatic Zika virus infection in the general population and in specific population groups from observational epidemiological studies.

## Methods

We used systematic review methods, including a meta-analysis.^8^^,^^9^ We registered the protocol on the International prospective register of systematic reviews (CRD42017059342)^10^ and followed the Preferred Reporting Items for Systematic Reviews and Meta-Analysis statement for reporting.^11^

### Inclusion criteria

We included general or specific population-based studies of participants of all ages and from any country: pregnant women, newborns and infants, children, adults, newborns with congenital abnormalities, and adults with Guillain‒Barré syndrome and other neurological diseases.

We included studies if exposure to Zika virus was identified, using molecular or serological methods. We used the Pan American Health Organization (PAHO),World Health Organization (WHO) guidelines for laboratory testing wherever possible.^12^^,^^13^ For a confirmed case these guidelines require: (i) presence of ribonucleic acid or Zika virus antigen in any specimen (serum, urine, saliva, tissue or whole blood) tested by reverse-transcriptase polymerase chain reaction method; or (ii) positive anti-Zika virus IgM antibodies and plaque reduction neutralization test for Zika virus titres ≥ 20 and four or more times higher than for other flaviviruses; and exclusion of other flavivirus; or (iii) in autopsy specimens, detection of the viral genome (in fresh or paraffin tissue) by molecular techniques, or detection by immunohistochemistry. In practice, this definition was often not used in studies, especially in earlier research. We therefore included studies using alternative definitions for positive laboratory testing if the definition was clearly stated. One alternative definition was the PAHO‒WHO guideline for probable cases: presence of Zika IgM antibodies, with no evidence of infection with other flaviviruses.^12^

We defined the primary outcome measure as percentage of all Zika virus-positive participants who were asymptomatic at the time of laboratory testing, or within 7 to 10 days of testing. The denominator was all participants who were Zika virus-positive. For the numerator, the PAHO‒WHO guidelines for signs and symptoms were used wherever possible, which require patients to have rash (usually pruritic and maculopapular) with two or more of the following signs or symptoms: fever, usually < 38.5 °C; conjunctivitis (non-purulent/hyperemic); arthralgia; myalgia; and/or periarticular oedema.^12^ In practice, not all studies used the PAHO‒WHO definition and we included studies using alternative definitions for symptoms if a clear definition was provided. Asymptomatic Zika virus-positive participants were those with no symptoms or with symptoms that did not meet the definition used for the particular study.

We included cross-sectional seroprevalence studies, cohort studies of pregnant women, cohort studies of newborns and infants, case‒control studies of Guillain‒Barré syndrome and other neurological diseases, case‒control studies of microcephaly and case series with at least 20 participants. The cut-off value of 20 participants for case series was chosen as a reasonable minimum number for which prevalence data can be reported. A cross-sectional seroprevalence study in the general population is the most appropriate design to determine the prevalence of asymptomatic Zika virus infection. However, to make use of the limited information that was available, we chose to include other study designs and other populations. Published and completed unpublished studies were eligible for inclusion. Data from ongoing studies were also eligible for inclusion when results from a representative sample were available.

Publications in English, French, Spanish or Portuguese were included. There was no restriction on year of publication.

We excluded studies in which having symptoms of Zika virus was a criterion for inclusion of participants in the study. This is because it would give a biased value for percentage asymptomatic of 100% solely due to the inclusion criteria. We also excluded studies where the percentage of participants who were asymptomatic could not be determined.

### Search strategy

The search strategy and keywords used are shown in Box 1. The titles and abstracts of these references were checked by one author against the inclusion criteria. Additional published articles were also identified through separate manual searches of PubMed® and revision of Zika virus article alerts by another author. The full text of any potentially relevant papers were checked by a second author and disagreements resolved by discussion and consultation with a third author. Papers excluded after review by a second reviewer and discussions between reviewers were detailed in a table, together with the reason for their exclusion. We also made contact (by email or in-person at key Zika virus meetings) with known research groups conducting cross-sectional studies of Zika virus. These groups were identified through the PAHO‒WHO Zika virus research platform, which includes research protocols that detail ongoing research related to the virus.^14^

Box 1 **Search strategy for the systematic review of the prevalence of asymptomatic Zika virus infection**

We searched PubMed®, Embase® and LILACS online databases from inception to date of search (4 November 2016, updated 7 March 2017 and 26 January 2018) using the term “zika” as text word for PubMed® and LILACS and “zika” as keyword (zika.mp) for Embase® (Ovid). References were imported into EndNote version X7 reference management software (Clarivate Analytics, Philadelphia, United States of America). The search was then limited using the terms: (cohort OR case control OR case-control OR series OR prospective OR retrospective OR longitudinal OR cross-sectional OR cross sectional OR observational OR transversal OR seroprevalence OR prevalence OR asymptomatic) in any field and then checked for duplicates.

### Data extraction

We extracted qualitative information into a Word version 14 table and quantitative data into an Excel version 14 spreadsheet (Microsoft Corporation, Redmond, USA). One author extracted the data and another author checked it: disagreements were resolved by discussion and consultation with a third author where necessary. We extracted the following data: country of study; region within the country; study design (cross-sectional, cohort, case‒control, case series); population (all ages, pregnant women, newborns and infants, newborns with congenital abnormalities, adults, adults with Guillain‒Barré syndrome); age range; period of study; definition of Zika virus positive according to laboratory tests; definition of symptomatic and asymptomatic Zika virus; preferential recruitment of participants with symptoms (yes/no); sample size calculation; and comments.

Quantitative data extracted included: response rate; total number of participants; total number classified as Zika virus positive; number of Zika virus-positive participants classified as symptomatic and as asymptomatic; and percentage of the total sample who were symptomatic at time of recruitment. For the cohort studies we used Zika virus-positive status at any time during the pregnancy (for studies of pregnant women) or any time during the study (for studies of newborns and infants). We extracted quantitative data for relevant subgroups where the data and sample size allowed, including for population subgroups and different definitions of Zika virus exposure.

### Quality assessment

The quality of the included studies was assessed independently by two authors using the critical appraisal checklist for prevalence studies, developed by The Joanna Briggs Institute.^8^ This tool includes the same dimensions as the Assessing Risk of Bias in Prevalence Studies tool,^15^ but was considered more useful for this review as it is applicable to a variety of study designs. The Joanna Briggs Institute tool also includes extra items related to sample size and subgroups. Disagreements were resolved by discussion and consultation with a third author where necessary.

### Analysis

We summarized the findings from the included studies in numerical and narrative tables. We conducted quality-effects meta-analysis using *MetaXL* version 5.3 (Ersatz, EpiGear International, Sunrise Beach, Australia) and the double arcsine transformation of prevalence.^16^^–^^18^ We assessed heterogeneity using the *Q* and *I^2^* statistics. We used Doi plots and the Luis Furuya‒Kanamori index to evaluate the presence of small-study effects, where asymmetry can indicate publication or other biases.^16^ A symmetrical mountain-like plot with values of the Luis Furuya-Kanamori index within ± 1 indicates no asymmetry; between ± 1 and ± 2 indicates minor asymmetry; and exceeding ± 2 suggests major asymmetry.^16^ Due to the high degree of heterogeneity in the results, we also checked whether the heterogeneity could be explained by population subgroups. The number of included studies was insufficient for testing multiple subgroups. We also tested the sensitivity of the results to excluding the largest study^4^ and to using the actual sample figure, rather than the population estimate reported by the authors that accounts for symptoms not attributable to Zika virus infection.

## Results

**Fig. 1:**
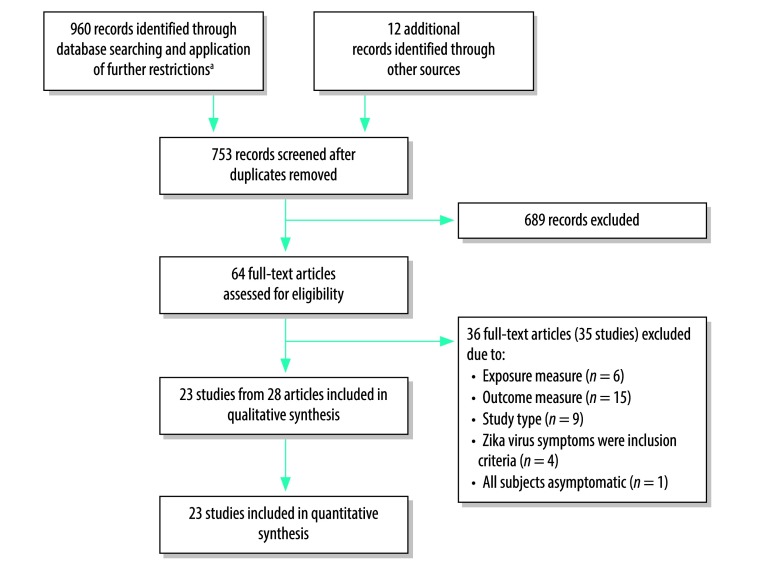
Flow diagram of selection of articles for the systematic review of the prevalence of asymptomatic Zika virus infection

^a^ Further restrictions were applied using Endnote reference management software (Clarivate Analytics, Philadelphia, United States of America; Box 1).

We identified a total of 960 records from database searches and another 12 records through other sources (Fig. 1). No unpublished or in-process studies were identified. After screening, we assessed 64 full-text articles for eligibility (Fig. 1) and excluded 36 articles^19^^–^^54^ for various reasons (Table 1). No studies were excluded due to language restrictions. A total of 23 studies from 28 articles met the inclusion criteria for the review (Table 2; available at: http://www.who.int/bulletin/volumes/96/6/17-201541).^4^^,^^5^^,^^55^^–^^80^

**Table 1:** Reasons for exclusion of studies from the systematic review of the prevalence of asymptomatic Zika virus infection

Study	**Exclusion category**	Reason for exclusion
Alvim et al., 2016^19^	Outcome measure	Percentage of participants with or without symptoms not reported
Brasil et al., 2016^23^^,^^24^	Exclusion criteria	Having symptoms was criterion for inclusion of participants
Brasil et al., 2016^22^	Exclusion criteria	Having symptoms was criterion for inclusion of participants
Carvalho et al., 2016^25^	Study type	Case series with < 20 cases (19 only)
De Paula-Freitas et al., 2016^27^	Exposure	No laboratory confirmation of exposure to Zika virus
Dirlikow et al., 2016^29^	Outcome measure	Percentage of participants asymptomatic not reported
Ferreira da Silva et al., 2016^31^	Exposure	No laboratory or molecular testing for Zika virus
Figueiredo et al., 2016^32^	Exclusion criteria	Having Zika virus symptoms was an inclusion criteria
Franca et al., 2016^33^	Study type	Very few participants tested for Zika virus either using PCR or serology (from email communication with corresponding author on 28 March 2017)
Hamer et al., 2016^36^	Outcome measure	Percentage of participants with or without symptoms not reported
Mani, 2016^40^	Study type	Summary of another study^33^ that was excluded due to very few participants undergoing laboratory testing
Melo et al., 2016^42^	Study type	Case series with < 20 cases (11 only)
Nah et al., 2016^44^	Outcome measure	Participants’ symptoms not reported. Modelling study
Sarno et al., 2016^47^	Exposure	No laboratory testing for Zika virus
Torres et al., 2016^50^	Outcome measure	Percentage of participants asymptomatic could not be measured as all Zika virus-positive participants had symptoms
Yakob et al., 2016^53^	Study type	No primary data presented
Araujo et al., 2017^20^	Outcome measure	Percentage of participants with or without symptoms not reported
Bierlaire et al., 2017^21^	Study type	Case series with < 20 cases (12 only)
Chow et al., 2017^26^	Outcome measure	Percentage of participants asymptomatic could not be determined as all enrolled participants were symptomatic
Eppes et al., 2017^30^	Exposure	Only 8 women had positive test results for Zika virus. Insufficient information to calculate percentage of participants with or without symptoms
Gonzalez et al., 2017^34^	Outcome measure	Percentage of participants with or without symptoms not reported
Griffin et al., 2017^35^	Exclusion criteria	Majority of children were selected for testing for Zika virus on the basis of having symptoms
Hancock et al., 2017^37^	Exposure	Exposure data reported for a period where all cases tested positive for Zika virus by real-time reverse transcription-PCR
Huits et al., 2017^38^	Study type	Only 6 of 31 travellers had confirmed Zika virus infection
Lee et al., 2017^39^	Outcome measure	Percentage of participants with or without symptoms not measured or reported
Marban-Castro et al., 2017^41^	Outcome measure	Insufficient information to decide whether study met inclusion criteria or to calculate percentage of participants with or without symptoms
Moreira et al., 2017^43^	Study type	Systematic review
Rac et al., 2017^45^	Outcome measure	Percentage of Zika virus-positive participants with or without symptoms not reported.
Salinas et al., 2017^46^	Outcome measure	Percentage of participants Zika virus-positive with or without symptoms not reported
Schaub et al., 2017^48^	Study type	Case series with < 20 cases (8 only)
Styczynski et al., 2017^49^	Outcome measure	Percentage of Zika virus-positive participants with or without symptoms not reported.
Tse et al., 2017^51^	Outcome measure	Percentage of participants with or without symptoms not reported. Likely that they were selected based on having symptoms
Uncini et al., 2017^52^	Outcome measure	Percentage of participants asymptomatic could not be measured as all Zika virus-positive participants had symptoms
Zambrano et al., 2017^54^	All asymptomatic	Data on symptoms not recorded at time of laboratory testing. All women were asymptomatic at enrolment
Delaney et al., 2018^28^	Exposure	Exposure to Zika virus tested in only a small proportion of participants

PCR: polymerase chain reaction.

**Table 2:** Characteristics of studies included in the systematic review of the prevalence of asymptomatic Zika virus infection

Study, author and year of primary reference^a^	Country or territory	Population	Study design	Definition of Zika virus positive	Definition of symptomatic Zika virus	Risk of bias score^b^
Duffy et al., 2009^4^	Federated States of Micronesia (Yap State)	General population	Cross-sectional	Evidence of recent infection: positive for IgM antibody against Zika virus by ELISA in serum	Defined as acute onset of generalized macular or papular rash, arthritis or arthralgia, or non-purulent conjunctivitis	8
Musso et al., 2014^55,56^	French Polynesia	Blood donors	Cross-sectional	Positive to Zika virus nucleic acid test in serum by real-time RT–PCR^c^	Not defined. Blood donors who were Zika-virus positive were telephoned and asked about “Zika fever-like syndrome” (rash, conjunctivitis, arthralgia) after their donation	7
Adams et al., 2016^57^	USA (Puerto Rico)	Pregnant women	Case series (surveillance)	Confirmed case: positive by RT–PCR in blood or urine. Presumptive case: positive Zika virus IgM by ELISA and negative dengue virus IgM by ELISA, or positive Zika virus by MAC-ELISA in a pregnant woman	Not defined	5
Araujo et al., 2016^58^	Brazil (metropolitan region of Recife)	Cases: neonates with microcephaly. Controls: live neonates without microcephaly, with no brain abnormalities or birth defects	Case–control	Positive by RT–PCR or IgM serum test of mothers and neonates	Not defined. Presence of maternal rash was reported	8
Cao-Lormeau et al., 2016^59^	French Polynesia	Cases: adults with Guillain–Barré syndrome. (Controls: excluded because no data on Zika symptoms were reported)	Case–control	Presence in serum of PRNT antibodies for Zika virus and anti-Zika virus IgG or IgM	Not defined. Described as recent history of viral syndrome before onset of neurological symptoms. Participants’ most commonly reported rash, arthralgia and fever	9
Dasgupta et al., 2016^60^	USA	Travellers;^d^ pregnant women travellers^d^	Case series (surveillance)	Confirmed case: detection of Zika virus RNA by RT–PCR or; anti-Zika IgM antibodies by ELISA with neutralizing antibody titres against Zika virus, at levels ≥ 4-fold higher than those against dengue virus	Defined as at least one of the following: fever, rash, arthralgia, or conjunctivitis	5
de Laval et al., 2016^61^	French Guiana	Travellers^d^	Cohort	Confirmed case: viral RNA detected by real-time PCR in blood or urine, or Zika virus IgM antibodies and neutralizing antibodies found in serum. Malaria excluded by thin and thick blood smears; dengue and chikungunya viruses excluded by blood real-time PCR	Not defined. All participants had cutaneous rash or other symptoms	3
Díaz-Menéndez et al., 2016^62,63^	Spain (Madrid; one hospital)	Travellers^d^	Case series	Confirmed case: positive microneutralization antibodies and/or positive RT–PCR for RNA in urine, blood, semen or amniotic fluid^e^	Not defined. Participants had one or more of: temperature > 38 °C, maculopapular rash, arthralgia, red eyes or headache	6
Leal et al., 2016^64^	Brazil (Pernambuco; one hospital)	Babies with microcephaly	Case series	Positive by Zika virus-specific IgM capture ELISA in cerebrospinal fluid	Not defined. Presence and timing of maternal rash during pregnancy was reported	4
Pacheco et al., 2016^65^	Colombia	Babies with possible microcephaly	Case series (surveillance)	Positive for Zika virus RNA in serum using RT–PCR and negative for syphilis, toxoplasmosis, other agents, rubella, cytomegalovirus and herpes virus tests, and normal karyotypes	Defined as fever and rash, plus at least one of the following symptoms: nonpurulent conjunctivitis, headache, pruritus, arthralgia, myalgia or malaise	6
Parra et al., 2016^66^	Colombia (Cucuta, Medellín, Neiva, Barranquilla and Cali; six hospitals)	Adults with Guillain–Barré syndrome	Case series	Definite case: positive for Zika virus RNA in blood, cerebrospinal fluid or urine by RT–PCR. Probable case: positive ELISA for antiflavivirus antibodies in cerebrospinal fluid, serum or both, but negative RT–PCR for Zika virus and for the four dengue virus serotypes	Defined as onset of systemic symptoms by Pan American Health Organization case definition	6
Adhikari et al., 2017^67,68^	USA (Dallas, Texas)	Pregnant women travellers^d^	Case series (screening)^f^	Probable case: positive by serum IgM test or real-time RT–PCR (serum or urine or both). Confirmation by serum PRNT^g^	Not defined. Participants’ symptoms included rash, fever, conjunctivitis and arthralgia	8
Aubry et al., 2017^69^	French Polynesia	General population, including schoolchildren	Cross-sectional	Positive for Zika virus IgG in blood by recombinant antigen-based indirect ELISA (schoolchildren) or in serum by microsphere immunoassay (general population)	Not defined. Participants were asked “whether they had clinical manifestations suggestive of past Zika infection”	6
Flamand et al., 2017^70^	French Guiana	Pregnant women	Cohort	Zika virus-positive by real-time RT–PCR in at least one blood or urine sample, or positive for Zika virus IgM antibodies in serum, irrespective of IgG results^h^	Defined as a clinical illness compatible with Zika virus in the 7 days before confirmation by RT–PCR or between the beginning of the outbreak and the date of laboratory diagnosis for IgM-positive cases. A compatible clinical illness was defined as at least one of the following symptoms: fever, a macular or papular rash, myalgia, arthralgia or conjunctival hyperaemia	9
Lozier et al., 2017^71^	Puerto Rico	General population (within 100 m radius of the residences of 19 index cases)	Cross-sectional (household-based cluster investigations)	Current infection: detection of Zika virus nucleic acid by RT–PCR in any specimen (serum, urine or whole blood). Recent infection: detection of anti-Zika virus IgM antibody by ELISA in serum. Recent flavivirus infection: detection of both anti-Zika virus IgM and anti-dengue virus IgM antibodies by ELISA in a serum specimen, in the absence of Zika virus or dengue virus nucleic acid detection (results were a subset of recent Zika virus infection). Zika virus positivity: evidence of current or recent Zika virus or flavivirus infection	Defined as presence of rash or arthralgia	7
Meneses et al., 2017^72^	Brazil	Babies with congenital Zika virus syndrome	Case series^f^	Zika virus-specific IgM tested by MAC-ELISA in cerebrospinal fluid. Positive results were followed by PRNT to confirm specificity of IgM antibodies against Zika virus and rule out cross-reactivity against other flaviviruses, including dengue	Defined as presence of symptoms related to a possible Zika virus infection during gestation: fever, maculopapular rash, arthralgia and conjunctivitis	4
Pomar et al., 2017^73,74^	French Guiana (Western part)	Pregnant women. Babies with congenital Zika virus syndrome	Case series (screening)^f^	Positive by RT–PCR (using the RealStar® Zika kit; Altona Diagnostics GmbH, Hamburg, Germany) in blood or urine or both, or by anti-Zika virus antibody detection using an in-house (National Referral Centre) IgM and IgG antibody-capture ELISA	Not defined. Participants’ symptoms were fever, pruritus, erythema, conjunctivitis, arthralgia or myalgia	6
Reynolds et al., 2017^5,75^	USA	Pregnant women	Case series (surveillance)^f^	Recent possible infection: based on presence of Zika virus RNA by nucleic acid test (e.g. RT–PCR) on any maternal, placental, fetal, or infant specimen (serum, urine, blood, cerebrospinal fluid, cord serum and cord blood); or serological evidence of recent Zika virus infection or recent unspecified flavivirus infection from a maternal, fetal or infant specimen (i.e. Zika virus PRNT titre ≥ 10 with positive or negative Zika virus IgM, and regardless of dengue virus PRNT titre). Infants with positive or equivocal Zika virus IgM were included, provided a confirmatory PRNT was performed on a maternal or infant specimen	Not defined	5
Rodo et al., 2017^76^	Spain	Pregnant women travellers^d^	Case series^f^	Not defined. Reported as confirmed by RT–PCR, or probable by positive Zika virus-IgM or positive Zika virus neutralization tests (specimen type not reported)	Not defined. 13/17 symptomatic pregnant women had a rash	1
Rozé et al., 2017^77^	France, Martinique	Adults with Guillain–Barré syndrome	Cohort	Recent infection: Zika virus nucleic acid detected by RT–PCR in any specimen (cerebrospinal fluid, urine and plasma); or serum antibodies to Zika virus detected by Zika virus MAC-ELISA, and negative IgM MAC-ELISA against dengue virus or positive for neutralizing antibodies against Zika virus	Not defined. Participants’ symptoms were described as “preceding arbovirus-like syndrome,” characterized by fever, headache, retro-orbital pain, nonpurulent conjunctivitis, maculopapular rash, arthralgia or myalgia	6
Shapiro-Mendoza et al., 2017^78^	United States Territories and freely associated States	Pregnant women. Babies with ≥ 1 birth defect	Case series (surveillance)^f^	Recent possible infection: based on presence of Zika virus RNA by nucleic acid test (e.g. RT–PCR) on any maternal, placental, fetal, or infant specimen (serum, urine, blood, cerebrospinal fluid, cord serum and cord blood); or serological evidence of recent Zika virus infection or recent unspecified flavivirus infection (i.e. Zika virus PRNT titre ≥ 10 with positive or negative Zika virus IgM, and regardless of dengue virus PRNT titre). Infants with positive or equivocal Zika virus IgM were included, provided a confirmatory PRNT was performed on a maternal or infant specimen (serum, urine, and cerebrospinal fluid)^i^	Defined as one or more signs or symptoms consistent with Zika virus disease: acute onset of fever, rash, arthralgia or conjunctivitis	5
Stone et al., 2017^79^	USA	Zika virus RNA-positive blood donors	Cohort	Blood compartments and body fluids (whole blood, plasma, urine, saliva and semen) were tested for Zika RNA by real time RT–PCR. Plasma samples were tested for Zika virus IgM and IgG antibodies (specimen type not reported)	Not defined. Participants developed “multiple Zika virus-related symptoms”	2
Shiu et al., 2018^80^	USA	Pregnant women	Case series (screening)	PRNT was performed if real-time RT–PCR or IgM in serum or urine was positive. Women with non-negative Zika virus IgM, Zika virus PRNT > 10 and dengue virus PRNT < 10 were considered to be infected with Zika virus. Women with IgM-positive tests, but with PRNT results not yet available were also included	Not defined. Participants had “documented symptoms suspicious for Zika virus infection”	7

ELISA: enzyme-linked immunosorbent assay; Ig: immunoglobulin; MAC-ELISA: IgM antibody capture enzyme-linked immunosorbent assay; RNA: ribonucleic acid; PRNT: plaque reduction neutralization test; RT–PCR: reverse transcription-polymerase chain reaction; USA: United States of America.

^a^ If a study had more than one reference, we awarded one reference the status of primary reference.

^b^ The risk of bias was measured using the critical appraisal checklist for prevalence studies developed by the Joanna Briggs Institute,^8^ which has a maximum score of 10. The risk of bias scores ranged from 1 to 9, with a mean score of 5.8.

^c^ A sample was considered positive when amplification showed a cycle threshold value < 38.5. However, to avoid false-negative results due to the pooling, each minipool showing a cycle threshold value < 40 with at least one primer-probe set was controlled by individual RT–PCR. Even if the two primers-probe sets did not react with the four dengue virus serotypes,^16^ the specificity of the amplified product from two donors whose blood was Zika virus-positive by RT–PCR was controlled by sequencing.^56^

^d^ Travellers were those with recent travel to or from a Zika-affected area.

^e^ A patient where the detection of RNA of Zika virus by means of a confirmed positive PCR (two positive PCRs designed with different genomic targets and similar sensitivity or in different aliquots of the same sample) was obtained, was considered as a confirmed case. The confirmation of positive cases by immunofluorescence tests requires positive results in microneutralization tests.^62^

^f^ The study was actually a cohort study but only the baseline data are used here.

^g^ Serum IgM assay was performed by Dallas County Health and Human Services for specimens collected > 2 weeks after travel in asymptomatic and symptomatic pregnant women, up to 9 months after return from travel. Presumptive positive or equivocal serum IgM specimens were forwarded to the United States Centers for Disease Control and Prevention for confirmatory PRNT testing. Serum real-time RT–PCR for Zika virus RNA was performed by Dallas County Health and Human Services on any specimen collected within 4 weeks of symptom onset or within 6 weeks of return from travel. In August 2016, following release of the interim guidance for urine testing and evaluation of pregnant women, the authors implemented real-time RT–PCR testing of subsequent urine specimens for pregnant women with presumptive positive or equivocal serum IgM.^68^

^h^ Serology was done using an in-house MAC-ELISA (based on whole virus antigens obtained in cell culture and on hyperimmune ascitic fluid) at each trimester of pregnancy. The sensitivity of the test was evaluated in sera from 71 patients with Zika virus infection confirmed by real-time PCR between day 5 and day 20 after symptom onset, was 87% and increased to more than 98% for sera sampled after day 7 from symptoms onset. The specificity was very low in sera from people with confirmed acute dengue virus infection, but increased to more than 80% for a panel of sera-negative samples for all tested arboviruses.

^i^ The use of PRNT for confirmation of Zika virus infection is not routinely recommended in Puerto Rico; dengue virus is endemic and cross-reactivity is likely to occur in most cases. In Puerto Rico, detection of Zika virus IgM antibodies in a pregnant woman, fetus or infant (within 48 hours after delivery) was considered sufficient to indicate recent possible Zika virus infection.

We found only three cross-sectional seroprevalence studies of the general population, which are considered to be the most appropriate design to measure prevalence. These included the original study of Yap State residents, Federated States of Micronesia, conducted in 2007,^4^ a study of the general population and schoolchildren in French Polynesia conducted in 2014–2015^69^ and a study in 2016 of the general population living near 19 index cases in San Juan, Puerto Rico.^71^ The majority of the studies were case series from population health surveillance programmes,^57^^,^^60^^,^^65^^,^^75^^,^^78^ systematic screenings of an at-risk population^68^^,^^74^ or hospital-based screenings of an at-risk population.^62^^,^^64^^,^^66^^,^^72^^,^^76^^,^^80^ A cohort design was used in four studies,^61^^,^^70^^,^^77^^,^^79^ a case‒control design in two studies,^58^^,^^59^ and a cross-sectional study of blood donors in one study^56^ (Table 2).

There was considerable variation in the methods of laboratory testing and the definitions of Zika virus positivity used in the studies (Table 2). Also, few studies offered a definition for symptomatic or asymptomatic. Sample sizes in studies varied from 30 to over 9000 (Table 3).

**Table 3:** Results of the systematic review of the prevalence of asymptomatic Zika virus infection

Study, primary reference^a^	Population or subgroup	Total no. of participants	No. classified as Zika virus positive	No. asymptomatic	% asymptomatic (95% CI)	Comments
Duffy et al., 2009^4^	General population: adjusted figures	6 892	5 005	4 086	82 (81–83)	Figures adjusted for the percentage of symptoms unlikely to be attributable to Zika virus infection and adjusted to total Yap State population (3+ years of age)
	General population: actual figures	(557)^b^	(414)^b^	(258)^b^	(62 (58–67))^b^	Actual figures from tested sample
Musso et al., 2014^56^	Blood donors	1 505	42	31	74 (59–86)	Bias towards asymptomatic participants
Adams et al., 2016^57^	Pregnant women	9 343	426	43	10 (7–13)	Confirmed cases only
Araujo et al., 2016^58^	Cases: babies with microcephaly	32	13	6	46 (20–74)	Symptoms were measured in mothers
	Controls: babies without microcephaly or birth abnormalities	62	0	0	0	Not included in meta-analysis because no babies were Zika virus positive
Cao Lormeau et al., 2016^59^	Adults with Guillain–Barré syndrome	42	42	4	10 (2–21)	NA
Dasgupta et al., 2016^60^	Travellers	1 199	169	0	0 (0–1)	Bias towards symptomatic patients
	Pregnant women travellers	3 335	28	7	25 (10–43)	Bias towards symptomatic patients. United States Centers for Disease Control and Prevention recommendations changed during study
de Laval et al., 2016^61^	Travellers	136	10	3	30 (5–62)	All co-travellers were screened
Díaz-Menéndez et al., 2016^62^	Travellers	185	13	2	15 (0–41)	Bias towards symptomatic patients. World Health Organization definition of symptoms was applied to data
Leal et al., 2016^64^	Babies with microcephaly	70	63	9	14 (7–24)	NA
Pacheco et al., 2016^65^	Babies with microcephaly	50	4	4	100 (61–100)	NA
Parra et al., 2016^66^	Adults with Guillain–Barré syndrome	42	17	0	0 (0–10)	Authors reported two definitions of Zika virus-positive: definite and probable. We used results from the definite definition
Adhikari et al., 2017^68^	Pregnant women travellers	547	29	24	83 (67–95)	All pregnant women who had recently travelled were screened
Aubry et al., 2017^69^	General population: schoolchildren	476	312	91	29 (24–34)	NA
	General population	896	251	123	49 (43–55)	NA
Flamand et al., 2017^70^	Pregnant women	3 050	573	440	77 (73–80)	NA
Lozier et al., 2017^71^	General population	367	114	65	57 (48–66)	Household-based cluster investigation around 19 index cases
Meneses et al., 2017^72^	Babies with congenital zika virus syndrome	87	87	21	24 (16–34)	Symptoms were measured in mothers during pregnancy
Pomar et al., 2017^74^	Babies with congenital Zika virus syndrome	124	9	3	33 (6–68)	Symptoms were measured in mothers during pregnancy
	Pregnant women	1 690	301	249	83 (78–87)	Tried to recruit a representative sample of all pregnant women
Reynolds et al., 2017^75^	Pregnant women	972	947	599	63 (60–66)	Zika virus-positive cases included women with possible recent Zika virus infection
	Pregnant women (diagnosis confirmed)	(972)^b^	(243)^b^	(102)^b^	(42 (36–48))^b^	Women with recent Zika virus infection confirmed by nucleic acid test
Rodo et al., 2017^76^	Pregnant women travellers	183	39	22	56 (40–72)	NA
Rozé et al., 2017^77^	Adults with Guillain–Barré syndrome	30	23	7	30 (13–51)	NA
Shapiro-Mendoza et al., 2017^78^	Pregnant women	2 549	2 549	966	38 (36–40)	Zika virus-positive included possible recent Zika virus infection
	Babies with ≥ 1 birth defect	122	122	41	34 (25–42)	Symptoms were measured in mothers
Stone et al., 2017^79^	Blood donors	50	50	22	44 (30–58)	NA
Shiu et al., 2018^80^	Pregnant women	2 327	67	53	79 (68–88)	Symptom information was missing for 19 women
**Total**	**NA**	**36 363**	**11 305**	**6 921**	**NA**	**NA**

NA: not applicable.

^a^ If a studied had more than one reference, we awarded one reference the status of primary reference. All study references are presented in Table 1.

^b^ These data are shown in parentheses because they do not contribute to the primary result but were used in sensitivity analyses.

Note: We searched for studies published from inception of the databases until 26 January 2018.

The risk of bias scores ranged from 1 to 9 out of a possible total of 10, with a mean score of 5.8 (Table 2). The most common limitations were: sample not clearly representative of the population (18 studies); response rate not reported, or large number of non-responders (19 studies); and not accounting for confounding factors or failure to identify subgroup differences (17 studies). The three cross-sectional seroprevalence studies of the general population had risk of bias scores between 6 and 8.

**Fig. 2:**
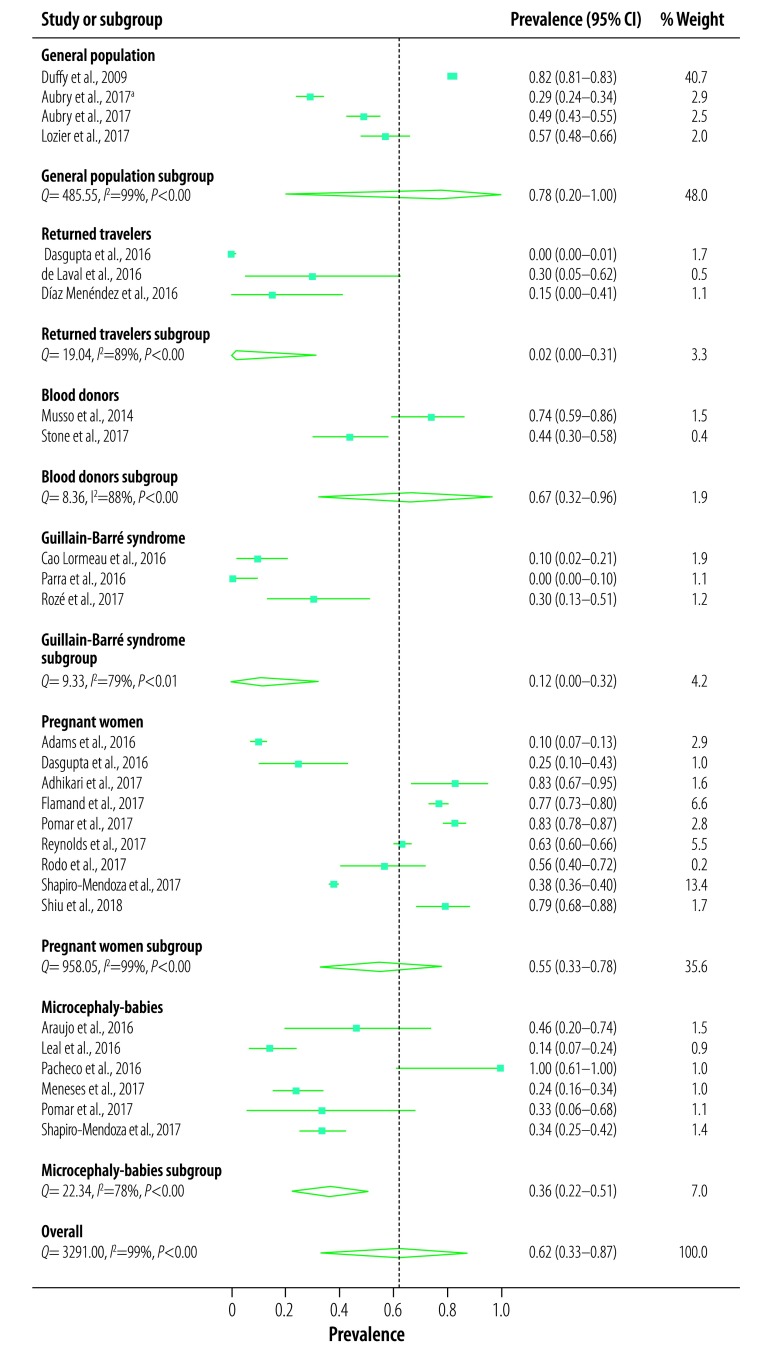
Prevalence of asymptomatic Zika virus infection in the systematic review of the literature

^a ^schoolchildren

The 23 studies included a pooled number of 11 305 participants positive for Zika virus, 6921 of whom were asymptomatic. Meta-analysis showed a combined prevalence of asymptomatic Zika virus of 61.8% (95% CI: 33.0–87.1%). However, there was substantial heterogeneity (*Q* = 3291, *P* < 0.001, *I^2^* = 99%), suggesting that the pooled prevalence is probably not a robust estimate. Analysis based on subgroups of the population (general population, returned travellers, blood donors, adults with Guillain‒Barré syndrome, pregnant women or babies with microcephaly) was not able to explain the heterogeneity (Fig. 2). There was also significant heterogeneity within all subgroups.

CI: confidence interval.

Notes: We searched for studies published from inception of the databases until 26 January 2018. The forest plot shows percentage of participants who tested positive for Zika virus and were asymptomatic. Prevalence was estimated from the quality effects model and using the double arcsine transformation of prevalence. The dotted line represents the combined prevalence found in the meta-analysis (0.62).

**Fig. 3:**
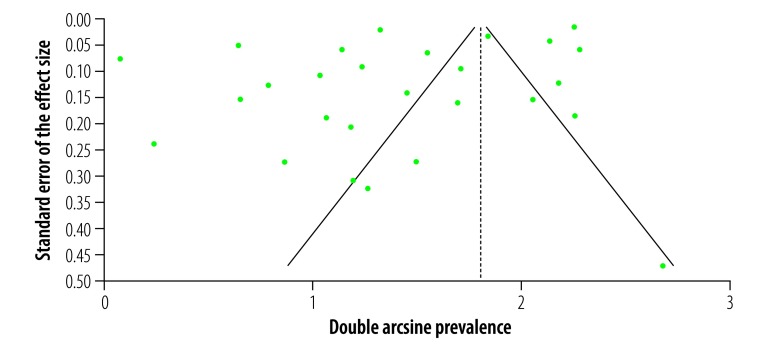
Funnel plot of publication bias in the systematic review of the prevalence of asymptomatic Zika virus infection

Note: The vertical line represents the combined effect size from the fixed effect meta-analysis

**Fig. 4:**
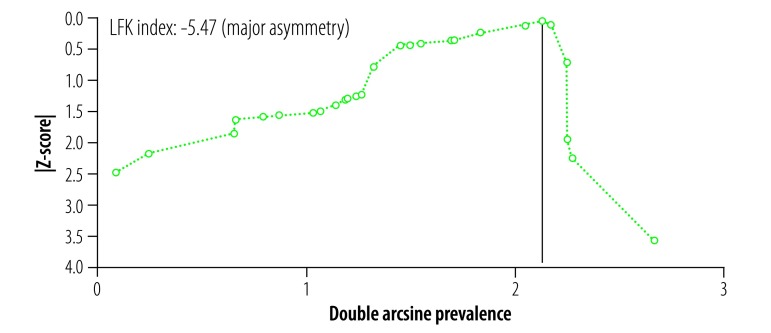
Doi plot of publication bias in the systematic review of the prevalence of asymptomatic Zika virus infection

Both the funnel plot (Fig. 3) and Doi plot (Fig. 4) showed major asymmetry. The most likely explanations for the asymmetry are selection bias, including publication bias, or true heterogeneity in the included studies.^81^ The largest study (population-adjusted sample: 6892; actual sample: 557)^4^ had a weight of 40.7% in the meta-analysis. Excluding this study completely removed the asymmetry (Luis Furuya-Kanamori index: 0.05) but not the heterogeneity (*Q* = 1484.5, *P* < 0.001, *I^2^* = 98%). The study’s exclusion also resulted in a substantial reduction in the pooled estimate to 45.2% (95% CI: 28.9–62.0%) and a narrowing of the confidence intervals. When the actual sample figures from this study^4^ were used instead of the population-adjusted figures the resulting pooled estimate was 46.5% (95% CI: 31.2–62.2%), with major heterogeneity (*Q* = 1537.1, *P* < 0.001, *I^2^* = 98%) but no asymmetry (Luis Furuya-Kanamori index: −0.57).

LFK: Luis Furuya-Kanamori.

Note: The vertical line represents the combined effect size from the quality effects meta-analysis.

## Discussion

Although we found 23 studies for this review, the high degree of heterogeneity in the studies made it difficult to form clear conclusions as to the true prevalence of asymptomatic Zika virus infection. Furthermore, subgroup analysis by population group was unable to explain the heterogeneity. While the prevalence of asymptomatic Zika virus infection appeared to be lower in returned travellers and adults with Guillain‒Barré syndrome, this could be due to the lack of representativeness of the samples, as those with symptoms are more likely to be tested.

The large variation in prevalence of asymptomatic Zika virus infection in the general population, which ranged from 29% (95% CI: 24–24%) in schoolchildren from French Polynesia^69^ to 82% (95% CI: 81–83%) in the general population of Yap State^4^ could be due to several reasons. One possibility could be the lack of representativeness of the French Polynesia sample as the response rate was not reported.^69^ A second possibility is that the population prevalence in Yap State was overestimated due to the method of assessing symptom status, which was done retrospectively and then adjusted for the percentage unlikely to be attributable to Zika virus infection.^4^ The high degree of sensitivity of the results to the removal of this study lends supports to this possibility. A third possibility is that differences in definitions of symptoms and criteria for Zika virus infection (including the diagnostic test used) could have led to differences in prevalence estimates. This possibility is supported by the lower prevalence of asymptomatic Zika virus infection in pregnant women with confirmed recent infection than in those with possible recent infection (42% versus 63%; Table 3) in the United States.^75^ Finally, the difference could be real.

The authors of a systematic review and meta-analysis of 55 influenza virus infection studies also found considerable heterogeneity in the proportion of asymptomatic infected persons.^82^ Despite the large number of studies, the heterogeneity could not be explained by the type of influenza, the laboratory tests used to detect the virus, the year of the study, or the location of the study.^82^ For Zika virus the amount and quality of the available evidence is insufficient to provide a single estimate of the prevalence of asymptomatic infection or to determine whether the heterogeneity found in this review is real.

In relation to the heterogeneity in prevalence, comparing two included studies that presented data on completed pregnancies from the United States Zika pregnancy registry and used similar surveillance methods is important.^75^^,^^78^ One study in the USA found an asymptomatic Zika virus infection prevalence of 63%;^75^ this is consistent with an earlier report of 61% from the same population,^5^ suggesting little variation over time. The other study was of completed pregnancies in United States Territories (American Samoa, Puerto Rico and United States Virgin Islands) and the Federated States of Micronesia and Marshall Islands^78^ and found a prevalence of asymptomatic Zika virus infection of 38%.^78^ If the difference is real or a result of differences in ascertainment of asymptomatic Zika virus infection is difficult to know. The registry is based on surveillance systems, which depend on testing in clinical practice and which can be affected by the care-seeking behaviour of the population. This raises the issue of the ability of surveillance systems to provide unbiased results for Zika virus research questions.^83^

Although we included population subgroups in our meta-analysis there were insufficient data to study the effect of demographic variables on the prevalence of asymptomatic Zika virus. While three of the included studies reported on age, sex or geographical differences in symptomatic infection,^69^^–^^71^ clear conclusions were not possible to make. 

A key strength of this review was the use of high-quality systematic review methods.^9^ Limitations of the review include the small number of studies found, especially cross-sectional seroprevalence studies, and the heterogeneity in the methods used across studies. The majority of studies included in the review were based on population health surveillance or screening programmes, rather than good-quality research studies. Furthermore, the included studies used various definitions of Zika virus positivity and rarely offered a definition for Zika virus symptom status. A variety of laboratory tests were used with varying degrees of validity, which can lead to potential misclassification error.^83^ A particular issue for Zika virus infection is the serological cross-reactivity of current IgM antibody assays with dengue virus, among other flaviviruses.^84^^,^^85^ The potential effect on the results is not known. In several studies there was also a bias towards inclusion of participants with symptoms due to the criteria for population surveillance or because symptomatic people are more likely to seek health care (e.g. travellers returning from Zika virus-endemic areas).

One clear finding from this review is that, given the current state of the evidence, it is not possible to give an accurate figure for the prevalence of asymptomatic Zika virus. Nor is it known whether the prevalence varies between populations or over time. Better-quality research is needed to estimate prevalence in the general population and in specific population groups. The use of standardized protocols developed by WHO and partners,^86^ particularly the protocol for the cross-sectional seroprevalence study of Zika virus infection in the general population,^13^ will be important in this regard. The protocol aims to standardize the diagnostic tests and definitions used, as well as encouraging consistent reporting.^13^^,^^86^ Use of the protocol will ensure results can be compared across regions and countries and help to improve the quality of the studies by minimizing bias.^86^ In this way the results of studies will better inform future public health surveillance and interventions.
